# Peripheral Artery Disease in Acute Ischemic Stroke Patients Treated With Endovascular Thrombectomy; Results From the MR CLEAN Registry

**DOI:** 10.3389/fneur.2020.560300

**Published:** 2020-10-07

**Authors:** France A. V. Pirson, Wouter H. Hinsenveld, Julie Staals, Inger R. de Ridder, Wim H. van Zwam, Tobien H. C. M. L. Schreuder, Yvo B. W. E. M. Roos, Charles B. L. M. Majoie, H. Bart van der Worp, Maarten Uyttenboogaart, Geert J. Lycklama à Nijeholt, Wouter J. Schonewille, Robert J. van Oostenbrugge

**Affiliations:** ^1^Department of Neurology, Maastricht University Medical Center, Maastricht, Netherlands; ^2^Department of Radiology, Maastricht University Medical Center, Maastricht, Netherlands; ^3^Department of Neurology, Zuyderland Medical Center, Heerlen, Netherlands; ^4^Department of Neurology, Amsterdam University Medical Center, Amsterdam, Netherlands; ^5^Department of Radiology and Nuclear Medicine, Amsterdam University Medical Center, Amsterdam, Netherlands; ^6^Department of Neurology and Neurosurgery, Brain Center, University Medical Center Utrecht, Utrecht, Netherlands; ^7^Department of Neurology and Radiology, University Medical Center Groningen, Groningen, Netherlands; ^8^Department of Radiology, Haaglanden Medical Center, The Hague, Netherlands; ^9^Department of Neurology, Sint Antonius Hospital, Nieuwegein, Netherlands

**Keywords:** peripheral artery disease, acute ischemic stroke, ischemic preconditioning, endovascular treatment, functional outcome

## Abstract

**Background and Purpose:** Though peripheral artery disease (PAD) is a well-known risk factor for ischemic events, better outcomes have been described in acute ischemic stroke patients with co-existing PAD. This paradoxical association has been attributed to remote ischemic preconditioning (RIPC) and might be related to better collateral blood flow. The aim of this study is to compare outcomes after endovascular thrombectomy (EVT) in acute stroke patients with and without PAD and to assess the relation between PAD and collateral grades.

**Methods:** We analyzed acute ischemic stroke patients treated with EVT for an anterior circulation large artery occlusion, included in the Dutch, prospective, multicenter MR CLEAN Registry between March 2014 and November 2017. Collaterals were scored on CT angiography, using a 4-point collateral score. We used logistic regression analysis to estimate the association of PAD with collateral grades and functional outcome, assessed with the modified Rankin Scale (mRS) at 90 days. Safety outcomes included mortality at 90 days, symptomatic intracranial hemorrhage, and stroke progression.

**Results:** We included 2,765 patients for analysis, of whom 254 (9.2%) had PAD. After adjustment for potential confounders, multivariable regression analysis showed no association of PAD with functional outcome [mRS cOR 0.90 (95% CI, 0.7–1.2)], collateral grades (cOR 0.85, 95% CI 0.7–1.1), or safety outcomes.

**Conclusion:** In the absence of an association between the presence of PAD and collateral scores or outcomes after EVT, it may be questioned whether PAD leads to RIPC in patients with acute ischemic stroke due to large vessel occlusion.

## Introduction

Patients with previous transient ischemic attacks seem to have smaller infarct sizes and better outcomes after subsequent cerebral infarction than patients without (1, 2). This phenomenon, in which brief periods of hypoperfusion offer protection in case of subsequent prolonged ischemia, is called ischemic preconditioning. The underlying etiology is not fully understood but seems to rely on systemic immunoreactivity and metabolic changes (3).

Animal studies have suggested that a similar neuroprotective effect occurs when hypoperfusion is induced in an organ other than the brain; so-called remote ischemic preconditioning (RIPC) (4, 5). In clinical setting, a randomized controlled trial in patients with ischemic stroke treated with intravenous thrombolysis demonstrated that RIPC induced by intermittently inflating a tourniquet on one of the limbs led to smaller perfusion deficits or DWI lesions (6). However, this trial did not show effect on clinical outcome at 3 months.

Peripheral artery disease (PAD) can be regarded as a chronic remote preconditioning process. So far there are conflicting results in studies with acute stroke patients on the relation between PAD and outcome. A small retrospective case-control study found that patients with ischemic stroke who had PAD had smaller infarct volumes, better functional outcomes, and a reduced risk of death (7). On the contrary, worse outcomes have been reported in stroke patients with low ankle-brachial index (ABI), which is a measure for PAD (8).

In a mouse model of ischemic stroke, RIPC led to better cerebral blood flow and the prevention of collateral artery collapse (9, 10). Whether pre-existent PAD affects the extensiveness of collaterals in humans is unknown. Since multiple studies have shown that higher collateral grades are associated with better functional outcome after endovascular treatment (EVT) in acute ischemic stroke, PAD may have a positive effect on outcome through better collateral blood flow (11–13).

The aim of our present study is (1) to investigate if pre-existent PAD is associated with better functional outcome in acute stroke patients treated with EVT, and (2) to investigate if PAD is correlated with collateral grades on CTA before EVT.

## Methods

### Study Design and Patients

The MR CLEAN Registry is a prospective, nationwide registry, in which data are collected from consecutive acute stroke patients treated with EVT in the Netherlands. The study protocol has been evaluated by the medical ethics committee of the Erasmus MC in Rotterdam, and permission to carry out the study as a registry was granted. Full methods of the MR CLEAN Registry have been reported previously (14). EVT consisted of mechanical thrombectomy, thrombus aspiration, or a combination of both. The method of EVT for each patient was left to the discretion of the treating physicians. For the present study, we used data of patients who underwent EVT from March 2014 up to November 2017 meeting the following inclusion criteria: groin puncture within 6.5 hours after symptom onset; age > 18; occlusion of intracranial carotid (ICA, ICA-T), middle (M1/M2), or anterior (A1/A2) cerebral artery, demonstrated by baseline CT angiography (CTA). Patients with missing information on PAD were excluded from assessment.

Source data will not be made available because of legislator issues on patient privacy, but detailed analytic methods and study materials, including log files of statistical analyses, will be made available to other researchers on reasonable request to the first author.

### Peripheral Artery Disease Assessment

PAD as well as other vascular risk factors were recorded as baseline parameters. These risk factors were mainly obtained from patient records, in which the diagnoses were recorded by the treating physicians. PAD could include vascular claudication or critical limb ischemia for which an intervention might have been performed.

### Imaging Assessment

All imaging was assessed by an imaging core laboratory, whose members were blinded to clinical findings, except for side of symptoms. Collateral status was graded on baseline CTA using a 4-point scale, with 0 for absent collaterals (0% filling of the occluded vascular territory), 1 for poor collaterals (>0% and ≤ 50% filling), 2 for moderate (>50% and <100% filling), and 3 for good collaterals (100% filling) (15). Reperfusion was scored on digital subtraction angiography (DSA) by the extended Thrombolysis in Cerebral Ischemia (eTICI) score (16), which ranges from grade 0 (no reperfusion) to grade 3 (complete reperfusion). Successful reperfusion was defined as eTICI 2B or higher. Patients of whom bi-directional view on final DSA was not available (missing lateral or anterior view), were excluded from further analysis.

### Outcome Assessment

The primary outcome measure was the modified Rankin Score (mRS) at 90 days, which is a 7-point scale ranging from 0 (no symptoms) to 6 (death) (17). A score of 2 points or less indicates functional independence. Secondary outcomes included functional independence, neurological deficit measured by the National Institute of Health Stroke Scale (NIHSS) at 24–48 h (18), and collateral grades. Safety outcomes included mortality at 90 days, symptomatic intracranial hemorrhage (sICH), and stroke progression. Intracranial hemorrhage was considered symptomatic if the patient had died or had deteriorated neurologically (a decline of at least four points on the NIHSS), and the hemorrhage was related to the clinical deterioration (according to Heidelberg criteria) (19).

### Statistical Analysis

Baseline characteristics were described using standard statistics. For the relation between PAD and functional outcome, we used multivariable ordinal logistic regression analysis to estimate the adjusted common odds ratio (acOR) for a shift toward a better functional outcome on the mRS. For the association between PAD and collaterals, we used univariable and multivariable ordinal logistic regression. In all multivariable analysis we adjusted for potential imbalances in prespecified prognostic variables: age, sex, NIHSS at baseline, hypercholesterolemia, hypertension, diabetes mellitus, smoking, previous myocardial infarction, previous stroke (only manifest ischemic stroke), ASPECTS on baseline non-contrast CT, reperfusion grade, intracranial atherosclerosis, and atherosclerotic stenosis of the symptomatic carotid artery.

All descriptive analyses include patients with complete data, while all regression models include all patients with imputed data. STATA (version 14.1) was used for all statistical analyses.

## Results

### Patient Characteristics

**Figure 1:**
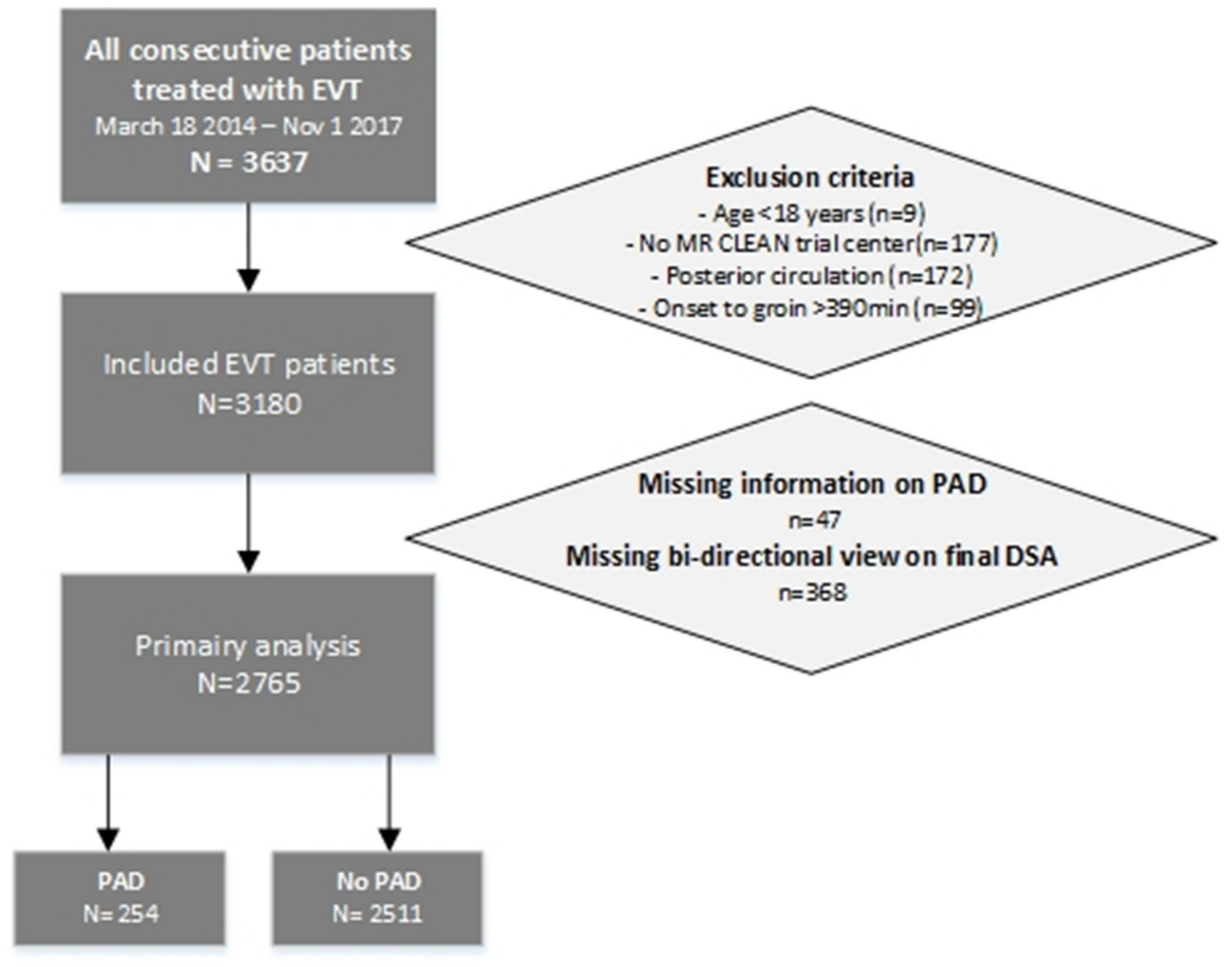
Flowchart patient selection.

Of the 3,180 patients in the MR CLEAN registry meeting all other inclusion criteria, 47 were excluded because of unrecorded information on PAD and 368 because of missing bi-directional view on final DSA, leaving 2,765 patients for the present *post-hoc* analysis. Of these, 254 (9.2%) had PAD (Figure 1).

Patients with PAD were older (median age 75 vs. 72, *p* < 0.02), and less often received intravenous thrombolysis (72 vs. 77%, *p* = 0.05) than patients without PAD (Table 1). Hypertension, myocardial infarction, hypercholesterolemia, diabetes mellitus, previous ischemic stroke, and smoking were more frequent in patients with PAD. The extent of early ischemic lesions at baseline was significantly different between groups, in favor of patients with PAD (median ASPECTS 9 (IQR 8–10) vs. 9 (IQR 7–10), *p* = 0.03). Patients with PAD were more likely to have intracranial atherosclerosis (76 vs. 59%, *p* < 0.01), and >50% atherosclerotic stenosis of the symptomatic carotid artery compared to patients without PAD (16 vs. 8%, *p* < 0.01). Successful reperfusion did not differ between groups (eTICI 2B-3 68 vs. 70%, *p* = 0.60).

**Table 1:** Baseline characteristics of ischemic stroke patients with peripheral artery disease (PAD) compared with no PAD.

**Total (*n* = 2,765)**	**PAD (*n* = 254)**	**No PAD (*n* = 2,511)**	***P*-value**
Age, y, median (IQR)	75 (66–80)	72 (61–80)	0.02
Male sex, *n* (%)	146 (57%)	1,304 (52%)	0.09
NIHSS, median (IQR)	16 (12–20)	16 (11–19)	0.74
Clinical localization: left hemisphere *n* (%)^*^	141/254 (56%)	1,320/2,509 (53%)	0.56
Mean (SD) systolic blood pressure (mm Hg)^†^	151 (26)	150 (25)	0.42
Intravenous alteplase treatment, *n* (%)	181/252 (72%)	1,936/2,503 (77%)	0.05
**Medical History**			
Atrial fibrillation, *n* (%)	66/249 (27%)	579/2,494 (23%)	0.24
Hypertension, *n* (%)	172/249 (69%)	1,233/2,469 (50%)	<0.01
Myocardial infarction, *n* (%)	71/250 (28%)	302/2,480 (12%)	<0.01
Hypercholesterolemia, *n* (%)	134/242 (55%)	672/2,22 (28%)	<0.01
Diabetes mellitus, *n* (%)	65/253 (26%)	382/2,505 (15%)	<0.01
Previous ischemic stroke, *n* (%)	60/251 (24%)	400/2509 (16%)	<0.01
Current smoking, *n* (%)	82/187 (44%)	515/1961 (26%)	<0.01
Pre-stroke modified Rankin Scale score, *n* (%)^‡^			<0.01
0	132 (52%)	1,701 (68%)	
1	41 (16%)	314 (13%)	
2	29 (11%)	172 (7%)	
>2	52 (20%)	324 (13%)	
Statin use, n (%)	153/245 (62%)	818/2,466 (33%)	<0.01
Antiplatelet use, n (%)	152/251 (61%)	706/2,486 (28%)	<0.01
Anticoagulation use, n (%)	82/250 (33%)	412/2,474 (17%)	<0.01
Antihypertensive medication use, n (%)	183/248 (74%)	1,277/2,471 (52%)	<0.01
Stroke etiology according to TOAST^§^			0.03
CE	44/125 (35%)	39/12,036 (33%)	
LAA	25/125 (20%)	145 /1,203(12%)	
Other, *e.g., dissection*	2/125 (2%)	54/1,203 (4%)	
Undetermined^||^	54/125 (43%)	608/1,203 (51%)	
**Radiological**			
ASPECTS on NCCT, median (IQR)^#^	9 (8–10)	9 (7–10)	0.03
Level of occlusion on non-invasive vessel imaging, n (%)^**^			0.54
ICA	12 (5%)	127 (5%)	
ICA-T	55 (22%)	490 (21%)	
M1	138 (56%)	1,417 (59%)	
M2	37 (15%)	334 (14%)	
Other *m3, A1, A2*	3 (1.2%)	18 (0.8%)	
Intracranial atherosclerosis on CTA, n (%)^††^	185 (76%)	1,416 (59%)	<0.01
Atherosclerotic stenosis at symptomatic carotid artery, *n* (%)[Table-fn TN7]			<0.01
No stenosis	74 (34%)	1,045 (47%)	
Stenosis <50%	111 (50%)	979 (44%)	
Stenosis >50%	35 (16%)	186 (8%)	
Reperfusion on DSA, *n* (%)^†^			
eTICI 2B-3	169 (68%)	1,708 (70%)	0.60
eTICI 2C-3	123 (48%)	1,157 (46%)	0.48

*CTA indicates computed tomographic angiography; ICA, intracranial artery; IQR, interquartile range; NCCT, non-contrast computed tomography; NIHSS, National Institutes of Health Stroke Scale; SD, standard deviation*.

* *15 patients underwent endovascular treatment without a definitive occlusion on CTA according to the core laboratory*;

‡ *n = 2,702, missing in 63 patients*;

§ *Registry part I data (march 2014–june 2016)*;

|| *undetermined, more than one potential cause, or negative/incomplete evaluation*;

# *n = 2669, missing in 96 patients*;

** *n = 2,631, missing in 134 patients*;

†† *n = 2,650, missing in 115 patients*;

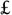
*n = 2,430, missing in 335 patients*,

† *n = 2,691; missing in 74 patients*.

### PAD and Outcomes

In univariable analysis, patients with PAD had worse functional outcome as compared with those without PAD (cOR 0.72, 95% CI 0.6–0.9) (Table 2). After adjustment for possible confounders, PAD was not associated with functional outcome (adjusted cOR 0.90 (95% CI, 0.7–1.2). There was no association between PAD and all other secondary outcomes: 90-days mortality (aOR 1.24, 95% CI 0.9–1.7), 24 h NIHSS (β 0.15, 95% CI −0.9–1.2), sICH (aOR 0.85, 95% CI 0.5–1.6), or stroke progression (aOR 0.99, 95% CI 0.6–1.6) (Table 2).

**Table 2:** Primary and secondary outcomes. Ischemic stroke patients with PAD compared with no PAD.

**Total (*n* = 2,765)**	**PAD *n* = 254**	**No PAD *N* = 2,511**	**Unadjusted OR (95% CI)**	**Adjusted OR (95% CI)**
**Radiological**				
Collateral score on CTA, *n* (%)^*^			0.79 (0.6–1.0)	0.85 (0.7–1.1)
Grade 0	15 (6%)	138 (6%)		
Grade 1	99 (41%)	832 (35%)		
Grade 2	96 (40%)	906 (39%)		
Grade 3	32 (13%)	471 (20%)		
**Clinical**				
mRS at 90d—median^†^	4 (2–6)	3 (2–6)	0.72 (0.6–0.9)	0.90 (0.7–1.2)
mRS score of 0–2 at 90 d—*n* (%)	84 (33%)	1,000 (40%)	0.75 (0.6–0.98)	0.85 (0.6–1.2)
NIHSS 24 h—median^§^	10 (4–16)	9 (3–16)	β 0.61 (−0.5–1.7)	β0 0.15 (−0.9–1.2)
**Safety Outcomes**				
Mortality at 90 days—*n* (%)	86 (36%)	630 (27%)	1.54 (1.2–2.0)	1.24 (0.9–1.7)
sICH	13 (5%)	133 (5%)	0.96 (0.5–1.7)	0.85 (0.5–1.6)
Progression of stroke	24 (9%)	241 (10%)	0.98 (0.6–1.5)	0.99 (0.6–1.6)

*OR, Odds ratios for PAD association with outcome variable, estimated with logistic regression analyses. Adjustments were made for age, sex, NIHSS at baseline, hypertension, diabetes mellitus, hypercholesterolemia, smoking, previous myocardial infarction, previous stroke, intracranial atherosclerosis, ASPECTS on baseline non-contrast CT, reperfusion grade, and atherosclerotic stenosis of the symptomatic carotid artery. CTA indicates computed tomographic angiography; IQR, interquartile range; mRS, modified Rankin Scale; NCCT, noncontrast computed tomography; NIHSS, National Institutes of Health Stroke Scale; sICH, symptomatic intracranial hemorrhage*.

* *n = 2,589, missing in 176 patients*;

† *n = 2,570, missing in 195 patients*;

§ *n = 2,495, missing in 270 patients*.

### PAD and Collateral Status

In patients with PAD poor collaterals were more frequent (grade 1: 41 vs. 35%), and good collaterals were less frequent (grade 3: 13 vs. 20%) than in those without PAD (Table 1). In univariable regression analysis on the four point collateral score, this translates to an OR of 0.79 (95% CI 0.6–1.0). After adjusting for potential confounding factors, there was no significant association of PAD with collateral status (aOR 0.85, 95% CI 0.7–1.1) (Table 2).

## Discussion

In this large nation-wide multicenter registry of patients with ischemic stroke treated with EVT, univariable analyses showed that PAD patients had worse collateral grades and worse functional outcomes than those without. However, after correction for possible confounders, there was no statistically significant association between PAD and functional outcome or collateral grades. As could be expected, there is a strong effect of the co-variables associated with PAD on functional outcome after EVT.

To our knowledge the association between PAD and collaterals in acute ischemic stroke has not been reported before. By comparison, ischemic stroke patients with significant carotid artery stenosis have better collateral flow on CTA than patients without carotid artery stenosis (20). In our study, patients with PAD more often had carotid artery stenosis of 50% or greater than patients without PAD, but their collateral grades were worse. This might be explained by a predominant effect of age or associated co-morbidities (21). Another contributing factor might be that nearly all patients in our study underwent single-phase CT-angiography. Assessment of collateral flow on singe phase CTA may lead to an underestimation of collaterals in case of delayed filling in combination with an early acquisition phase (12, 22). In our case, significant carotid artery stenosis could have therefore disproportionally affected the relation between PAD and collateral status through delayed intracerebral filling.

We found no association between PAD and functional outcome after ischemic stroke, in contrast to a previous case-control study (7). In that study acute stroke patients with PAD had a very low NIHSS at presentation (47% NIHSS 1–4). Our study concerns a distinct patient population with proven large vessel occlusions causing more severe strokes resulting in a rather high NIHSS and possibly less dispersion in post-stroke mRS. The influence of PAD may therefore be diminished in patients treated with EVT due to stroke severity. Another explanation might be that physicians treat less aggressively in case of persistent neurologic deficit after EVT and concomitant severe co-morbidity including PAD. With this self-fulfilling prophecy, a possible positive effect of PAD on outcome may therefore not have been investigated properly. A previous study found worse outcome after stroke in patients with low ankle-brachial index (ABI) (8). Our study did not measure ABI to determine severity of PAD. It is possible to have missed an association between severe PAD and outcome if the patients in our cohort would have had mainly moderate-to-high ABI.

The strength of our study is the large patient sample. All outcome measures have been collected prospectively according to protocol and independent of our present research question.

Nevertheless, there are limitations. First, there was no standardized assessment for peripheral artery disease which may have led to different judgment between centers or between physicians. Also, we were not able to assess the ABI in our patients. Since ABI is more reliable in identifying PAD than determination based on clinical judgment, we might have over- or underdiagnosed PAD in our cohort of ischemic stroke patients. However, previous research on PAD in stroke patients showed a comparable prevalence of around 10% (8, 23).

A second limitation concerns the presence of intracerebral atherosclerosis in our patients. Peripheral artery disease can be isolated in the lower limbs, but might also occur concurrently in the coronary arteries or cerebral arteries (24, 25). The extent of cerebral vessel atherosclerosis is associated with white-matter loss and lacunar infarctions and may therefore influence functional outcome. Although we adjusted our analysis for the presence of intracranial atherosclerosis, our model could not be corrected for the extent or specific location of this condition.

## Conclusion

In summary, our study showed no association between PAD and outcome after EVT for acute ischemic stroke nor an independent association between PAD and collateral blood flow. As such, we could not confirm the occurrence of RIPC in PAD patients with acute ischemic stroke. Future studies with more standardized measurement of PAD are recommended.

## Data Availability Statement

Source data will not be made available because of legislator issues on patient privacy, but detailed analytic methods and study materials, including log files of statistical analyses, will be made available to other researchers on reasonable request to the first author. Requests to access the datasets should be directed to fav.pirson@mumc.nl.

## Ethics Statement

The studies involving human participants were reviewed and approved by medical ethics committee of the Erasmus MC in Rotterdam. Written informed consent for participation was not required for this study in accordance with the national legislation and the institutional requirements.

## Author's Note

MR Clean Registry Investigators are included in Supplementary Material.

## Author Contributions

FP analyzed the data and wrote the first draft of the manuscript. All other authors critically reviewed the manuscript for intellectual content, read, and approved the final version of the manuscript.

## Conflict of Interest

Erasmus MC received funds from Stryker by Diederik Dippel, Aad van der Lugt, and from Bracco Imaging by Diederik Dippel. Amsterdam UMC received funds from Stryker for consultations by CM, YR, and Olvert Berkhemer. Maastricht UMC received funds from Stryker and Cerenovus for consultations by WZ. UMC Utrecht received funds from Bayer, Boehringer Ingelheim, and LivaNova for consultation by BW. The remaining authors declare that the research was conducted in the absence of any commercial or financial relationships that could be construed as a potential conflict of interest.

## Abbreviations

ABI: ankle-brachial index
acOR: adjusted common odds ratio
ASPECTS: Alberta stroke programme early CT score
CTA: CT angiography
DSA: digital subtraction angiography
eTICI: extended Thrombolysis in Cerebral Ischemia
EVT: endovascular treatment
IQR: interquartile range
LVO: large vessel occlusion
MR CLEAN: Multicenter Randomized Clinical Trial of Endovascular Treatment for Acute Ischemic Stroke in the Netherlands
mRS: modified Rankin Scale
NCCT: non-contrast CT
NIHSS: National Institutes of Health Stroke Scale
RIPC: Remote Ischemic Preconditioning
sICH: symptomatic intracranial hemorrhage
TOAST: Trial of Org 10172 in Acute Stroke Treatment.
